# Effects of Different Land Use Patterns on Soil Water in Loess Hilly and Gully Regions of China

**DOI:** 10.3390/plants12010021

**Published:** 2022-12-20

**Authors:** Min Tang, Chao Zhang, Xiaodong Gao, Pute Wu

**Affiliations:** 1 College of Hydraulic Science and Engineering, Yangzhou University, Yangzhou 225009, China; 2 Institute of Water-Saving Agriculture in Arid Areas of China, Northwest Agriculture and Forestry University, Yangling, Xianyang 712100, China

**Keywords:** loess hilly region, land use, soil water, grey relational analysis

## Abstract

Soil water is a major barrier to ecological restoration and sustainable land use in China’s Loess Hilly Region. For the restoration of local vegetation and the optimal use of the region’s land resources, both theoretically and practically, it is essential to comprehend the soil water regimes under various land use types. The soil water content in the 0–160 cm soil profile of slope cropland, terraced field, jujube orchard, and grassland was continuously measured using EC-5 soil moisture sensors during the growing season (May–October) in the Yuanzegou catchment in the Loess Hilly Region to characterize the changes in soil water in these four typical land use types. The results showed that in both years of normal precipitation and drought, land use patterns varied in seasonal variability, water storage characteristics, and vertical distribution of soil water. In the dry year of 2015, the terraced field effectively held water. During the growing season, the 0–60 cm soil layer’s average soil water content was 2.6%, 4.2%, and 1.8% higher than the slope cropland, jujube orchard, and grassland, respectively (*p* < 0.05), and the 0–160 cm soil layer’s water storage was 43.90, 32.08, and 18.69 mm higher than the slope cropland, jujube orchard, and grassland, respectively. The average soil water content of the 0–60 cm soil layer in the jujube orchard was 2.9%, 3.8%, and 4.5% lower than that of slope cropland, terraced field, and grassland, respectively, during the normal precipitation year (2014) (*p* < 0.05). Only 35.0% of the total soil water storage was effectively stored in the 0–160 cm soil layer of the jujube orchard during the drought year. There was a significant difference in the grey relational grade between the soil water in the top layer (0–20 cm) and the soil water in the middle layer (20–100 cm) under different land use types, with the terraced field having the highest similarity degree of soil water variation trend, followed by grassland, slope cropland, and jujube orchard. Slope croplands in the study region may be converted into terraced fields to enhance the effective use of rainfall resources and encourage the expansion of ecological agriculture. Proper water management practices must be employed to reduce jujube tree water consumption and other wasteful water usage in order to guarantee the jujube orchard’s ability to expand sustainably. This would address the issue of the acute water deficit in the rain-fed jujube orchards in the Loess Hilly Region.

## 1. Introduction

Water is a carrier of nutrient circulation and movement in the soil system [1], and the most active part of the water cycle in a watershed [2], affecting plant development, environmental restoration, and the rational distribution and effective use of water resources [3,4]. The dry and semi-arid loess hilly region is noted for its scant vegetation [5], severe soil erosion [6], fragile ecological environment, and difficult regional vegetation regeneration. The biggest barrier to plant growth, ecological rebound, and sustainable land use is soil water [7]. Long-term, intense population pressure has caused illogical land use in the region, which has led to an overuse of soil water and the development of a dry soil layer that is challenging to utilize sustainably [8]. Since the national program to convert cropland to forest in 1999, the Loess Plateau region’s land use structure has changed, and the land resource use has been varied. A thorough knowledge of the soil and water conditions of various land use types and their changing patterns is essential for the effective use of land resources, as well as for the restoration of vegetation and optimization of the region’s land use structure.

China has conducted much research in recent years on the impacts and dynamics of soil water in loess hilly areas under various land use practices [9,10,11,12]. In late August 2014, Lan et al. measured the soil water content using an artificial soil sample collection and drying method. They then studied the differences in soil water characteristics in 0–20 m soil profiles under representative vegetation types in the Mizhi artificial economic forest, Shenmu farmland-to-forest converted lands, and Yulin Yuyang windbreak and sand-fixing forest [13]. The impacts of various land use patterns on the water distribution and storage properties of deep soil profiles and ecological environment were examined in the loess hilly area. In order to study the spatial variation of soil water caused by land use patterns, Wang et al. collected soil samples with a 5 cm inner diameter soil auger at 0–300 cm depth in woodland, shrubland, grassland, and farmland in the north-south sample zone of the Loess Plateau from July to August 2014 and determined their mass water content by drying method [14]. Most domestic research reports on the impact of different land use types on soil water in the Loess Plateau summarize short time periods or specific events as general patterns, and the majority of soil water analysis data in the existing studies come from the drying method, time domain reflectometer (TDR), neutron meter method, and other artificially measured soil water content at regular intervals, lacking long-term continuous positioning measurements of soil water and study of inter-annual seasonal fluctuation. In this study, four typical land use types—slope cropland, terraced field, jujube orchard, and grassland—were investigated in the small watershed of Dianzegou, Qingjian County, Loess Hilly Region. The patterns of soil water change and the characteristics of water storage under different land use types during normal and dry years were systematically examined. This paper offers recommendations on soil water management for various land use types based on research findings, in an effort to provide a theoretical foundation for the efficient utilization of available natural rainfall resources and the best distribution of land use and ecological restoration in the Loess Hilly Region.

## 2. Materials and Methods

### 2.1. Overview of the Study Area

**Figure 1:**
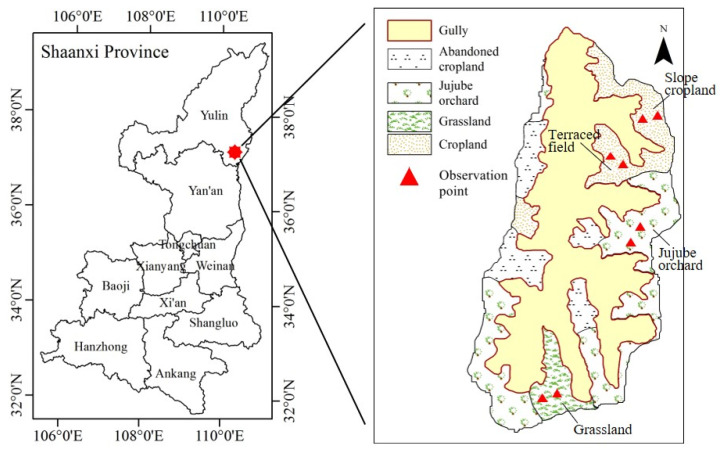
Location of the study area and distribution of experimental land use types.

**Figure 2:**
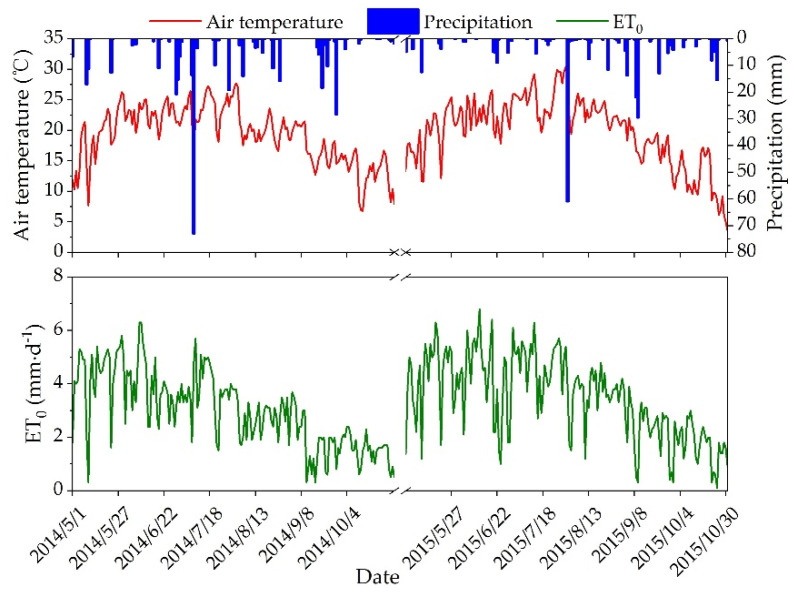
Precipitation, air temperature, and daily reference crop evapotranspiration (ET_0_) during the 2014 and 2015 growing seasons.

The study area is located in the Yuanzegou watershed of Dianzegou Town, Qingjian County, Shaanxi Province, China (37°14′ N, 110°21′ E; Figure 1), slightly to the north of the core Loess Plateau, with a watershed area of 0.58 km^2^. With a characteristic hilly and gully landscape, the region has a dispersed topography and a wide range of slopes, with around 70% of them lying between 17° and 34°. The region has a mild continental monsoon climate, with an annual average air temperature of 8.6 °C and monthly extremes of −6.5 °C in January and 22.8 °C in July. The average amount of precipitation each year is 505 mm, which is irregularly distributed throughout the year and largely concentrated from July to September. The majority of this precipitation is heavy rainfall, with substantial interannual fluctuation. The soil type in the research region is loessial, which has a loose texture, a high infiltration capacity, and a poor resistance to erosion. It was created from loess parent material. The field capacity and wilting point are 25% and 7%, respectively (volumetric water content, similarly henceforth). Due to the high intensity of land use, the watershed’s natural vegetation has been severely degraded, and the reclamation index is high, with land use types such sloping fields, terraces, jujube orchards, and grasslands. The year 2014 was a normal year with 377.4 mm of precipitation falling during the growing season (May to October) (precipitation declines within 10% of the annual average) [15]. Only 289.2 mm of precipitation fell throughout the growing season of 2015, making it a dry year (precipitation decreased by more than 10% from the annual normal) [15]. Figure 2 illustrates the precipitation, air temperature, and daily reference crop evapotranspiration (ET_0_) for the 2014 and 2015 growing seasons.

### 2.2. Experimental Design

Four representative land use types—slope cropland, terraced field, jujube orchard, and grassland—were chosen as experimental plots on the loess hilly slopes (Figure 1). All four land use types had slopes that were around 20° and in similar directions. The slope cropland had been cultivated for more than three decades, and the crop system consisted of a monoculture of soybeans (*Glycine max* (Linn.) Merr.), which were predominantly planted in late April and harvested in early October. Maize (*Zea mays* L.), which was sown in May and harvested between late September and early October, was the primary crop grown on the terraced field. Plant spacing for the 12-year-old “Lizao” cultivar of jujube (*Ziziphus jujuba* Mill.) was 2 m × 3 m. Average jujube tree height was (2.25 ± 0.28) m, and average crown width was (1.98 ± 0.38) m. The slope cropland, terraced field, and jujube orchard were all maintained using traditional farming techniques. The grassland was a naturally occurring barren grassland dominated by Artemisia gmelinil, thatch grass, and Lespedeza davurica. Table 1 displays the fundamental physical properties of the soil under different land use types. The experiment was conducted entirely under arid conditions, with only naturally occurring precipitation serving as a supplement.

### 2.3. Measurement of Soil Water, Precipitation, and Air Temperature

It has been demonstrated that maize’s roots may extend as deep as 160 cm [16], hence this article focuses on the soil water in the root zone of plants from 0 to 160 cm for each land use type. Two sets of automated soil moisture monitoring devices were installed at 10 m intervals along the same contour in the middle of the slope cropland, jujube orchard, and grassland, as well as the higher and lower portions of the terraced field. The monitoring equipment was installed in April 2014 for the terraced field, jujube orchard, and grassland; however, due to shipping issues, the slope cropland’s monitoring equipment wasn’t put in place until around a month later. An EC-5 soil moisture sensor (Decagon Devices Inc., Pullman, WA, USA) with an accuracy of ±3% VWC and a resolution of 0.25% VWC was used to measure the volumetric water content of the soil. In the sloped cropland and terraced field, monitoring probes were buried between crop rows, and the monitoring site in the jujube orchard was about 30 cm from the trunk of the jujube trees. During the growing season of 2014–2015 (May to October), soil water was monitored every 10 min at depths of 10, 20, 60, 100, and 160 cm. The soil water content at a particular depth was characterized by averaging soil water data from the same layer at two monitoring sites for each land type.

An AR5 automatic weather station was placed approximately 100 m away from the experimental site, allowing for long-term monitoring of meteorological factors such precipitation, wind speed, air pressure, solar radiation, air humidity, and air temperature in the study area.

### 2.4. Data Processing

#### 2.4.1. Soil Water Storage

Soil water storage (*SWS*, mm) is the amount of water kept in a certain amount of soil thickness, and it is calculated as follows [17].

(1) $$SWS=\sum_{i=1}^{n}\left(\theta_{i}\times h_{i}\right)$$

where *θ_i_* is the soil volumetric water content (in cm^3^·cm^−3^) of the *i*-th layer (out of a total of *n* layers) and *h_i_* is the thickness of this soil layer (mm).

#### 2.4.2. Soil Water Deficit

Soil water deficit (*D*, mm) is calculated as follows [18].

(2) $$D=\sum_{i=1}^{n}\left(SWS_{FC}-SWS_{i}\right)$$

where *FC* is the field capacity (cm^3^·cm^−3^), *SWS_FC_* is the soil water storage equivalent to *FC* (mm), and *SWS_i_* is the actual water storage of the *i*-th soil layer (mm).

#### 2.4.3. Effective Soil Water Storage

Effective water content is the amount of soil water that exists between the field capacity and the wilting point. The portion of the soil water content below the wilting point and above the field capacity is ineffective. Thus, effective soil water storage (*ESWS*, mm) is stated as follows [19].

(3) $$ESWS_{i}=\{\begin{array}{c} SWS_{i}-SWS_{WM}(\theta_{i}<FC)\\ SWS_{FC}-SWS_{WM}(\theta_{i}\geq FC) \end{array}$$

(4) $$ESWS=\sum_{i=1}^{n}ESWS_{i}$$

where *ESWS_i_* is the effective soil water storage in the *i*-th soil layer (mm) and *SWS_WM_* is the soil water storage related to the wilting point (mm).

#### 2.4.4. Grey Relational Analysis

Grey interconnect describes ambiguous connections between things, as well as unsure connections between system components and between system factors and principal behavior. By examining the geometric relationship of data series, the grey relational analysis method identifies the closeness of various components in a system (grey relational grade) as its guiding principle. The grey relational grade between them increases with the geometric closeness of the sequence curves [20]. In the past, most conventional statistical analysis or other analytic methods were utilized in systematic analyses of complex systems. In contrast to the former, the grey relational analysis method has the following advantages: Because grey relational analysis is based on component trend, sample size requirements are low and conventional distributional criteria are not essential. The calculation is quite simple, and the results agree with those of the qualitative analysis. Therefore, grey relational analysis is a simple and reliable analytical method [21].

Prior to performing a grey relational analysis, it is required to determine the reference series. After that, it is necessary to assess how closely the other series resembles the reference series in order to compare them and reach further reasonable conclusions. If *X*_0_ ={*X*_0_(*k*)|*k* = 1, 2, ..., *n*} is the reference series (also known as the parent series) and *X_i_* = {*X_i_* (*k*)|*k* = 1, 2, ..., *n*}(*i* = 1, 2, ..., *m*) is the comparison series (also known as the child series), then the relational coefficients of *X_i_*(*k*) and *X*_0_(*k*) may be found using the following formulas.

(5) $$\varepsilon_{i}(k)=\frac{\min_{i}\min_{k}\left|x_{0}(k)-x_{i}(k)\right|+\rho \max_{i}\max_{k}\left|x_{0}(k)-x_{i}(k)\right|}{\left|x_{0}(k)-x_{i}(k)\right|+\rho \max_{i}\max_{k}\left|x_{0}(k)-x_{i}(k)\right|}$$

The lower the resolution factor, represented byρ, the greater the resolution. The general range of ρ is [0, 1], and ρ = 0.5 is often used. |*x_0_*(*k*) − *x_i_*(*k*)| denotes the absolute difference between *X_0_* and the *k*-th indicator of *X_i_*; and $\min_{i}\min_{k}\left|x_{0}(k)-x_{i}(k)\right|$ and $\max_{i}\max_{k}\left|x_{0}(k)-x_{i}(k)\right|$ reflect, respectively, the two-level minimum and maximum discrepancies. Consequently, the relational coefficient between *X_i_*(*k*) and *X_0_*(*k*) can be determined as follows.

(6) $$\varepsilon_{i}=\left\{\varepsilon_{i}(k)|k=1,2,...,n\right\}$$

From the calculation of the relational coefficient, the value of the relational coefficient between each comparison series and the reference series at each point is obtained; however, the results are numerous, the information is too dispersed, making comparisons challenging; thus, it is necessary to focus the relational coefficient of each comparison series at each point on a value, this value being the grey relational grade *r*(*x*_0_, *x_i_*) between the comparison series and the reference series, usually abbreviated as *r_i_*. The formula for calculating the grey relational grade using the mean method is as follows.

(7) $$r_{i}=\frac{1}{n}\sum_{k=1}^{n}\varepsilon_{i}(k)$$

The grey relational grade of soil water reflects the cumulative effect of the soil water-influencing factors. In this study, soil water with a high grey relational grade can be utilized as a benchmark for comparing soil water across different land types and as a foundation for choosing soil water for comparison in other watersheds.

### 2.5. Data Analysis

One-way analysis of variance (ANOVA) with Tukey’s post-hoc test was performed to separate means at a significance level of *p* < 0.05.

## 3. Results

### 3.1. Variations in Soil Water Dynamics for Different Land Use Types

Approximately 90% of the total number of soybean roots are located in the 0–45 cm soil layer [22]. Approximately 95% of maize roots are dispersed in the 0–60 cm soil layer, whereas less than 2% are dispersed under 80 cm [23]. The bulk of the fine roots of jujube are situated in the 0–60 cm soil layer, and the root length density decreases with increasing soil depth below 60 cm [24]. On the Loess Plateau, natural wasteland grass roots are largely spread in the 0–60 cm soil layer. The roots of Artemisia gmelinil-dominated natural grasslands on the Loess Plateau are mostly dispersed in the 0–60 cm soil layer [25]. The soil profile was separated into a dense root layer (0–60 cm) and a sparse root layer (60–160 cm) based on the root distribution characteristics of the plants in each land use type.

**Figure 3:**
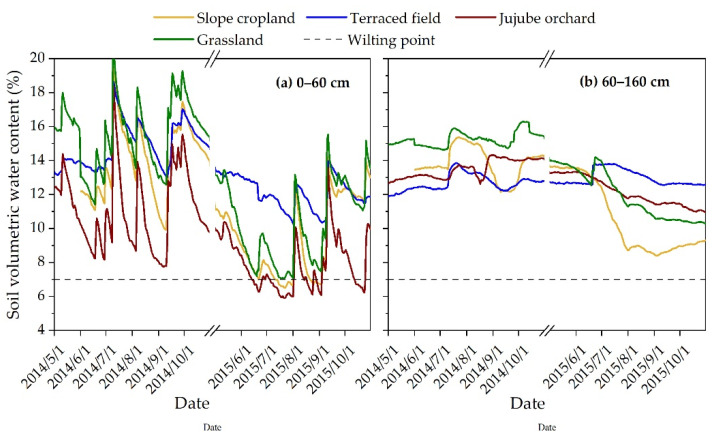
Variations in the daily average soil water content of the 0–60 cm (**a**) and 60–160 cm (**b**) soil layers during the growing season for various land uses. The daily mean soil water content of a soil layer for all land uses was calculated by averaging the soil water data collected by the EC-5 soil moisture sensor in a soil layer over one day.

Due to the combined impacts of precipitation, soil evaporation, and water absorption by plant roots, the soil water content of the dense root layer under each land use type changed considerably and belonged to the seasonal fluctuation layer, as seen in Figure 3a. Throughout the 2014 growing season, the soil water content in the dense root layer under all land types displayed peaks and valleys. The average soil water content in jujube orchard (11.2%) throughout the growing season was 2.9%, 3.8%, and 4.5% lower than in slope cropland, terraced field, and grassland, respectively (*p* < 0.05). In 2015, the soil water content in the dense root layer of each land type dropped throughout the early stages of the growing season, especially in jujube orchards, where rainfall was inadequate to provide soil evapotranspiration. Consequently, the soil water content in the dense root layer of the jujube orchard was lower than the wilting point during the full fruit stage, generating a seasonal low humidity area. Beginning in August 2015, the soil water content in the dense root layer under all land types started to recover as a result of the rainfall supplement, although the degree of recovery was modest. During the 2015 growing season, the soil water content in the dense root layer of terraced field was significantly higher than that of the other three land types (*p* < 0.05), with the average soil water content (12.2%) being 2.6%, 4.2%, and 1.8% greater than that of slope cropland, jujube orchard, and grassland, respectively. The rainfall had little influence on the soil water at the sparse root layer, and the soil water content progressively altered (Figure 3b). The soil water in a terraced field was not considerably affected by precipitation or drought. In the research region, the total rainfall for the 2014 and 2015 growing seasons was 377.4 and 289.2 mm, respectively (Figure 2). Consequently, the soil water content of the dense root layer and sparse root layer across all land types in 2015 was considerably lower than in 2014 (*p* < 0.05) (Figure 3). During the 2015 growing season, the average soil water content of the sparse root layer of terraced fields (13.0%) was 2.3%, 0.8%, and 1.0% more than slope cropland, jujube orchard, and grassland, respectively (*p* < 0.05) (Figure 3b).

### 3.2. Soil Water Characteristics in the Profile for Different Land Uses

**Figure 4:**
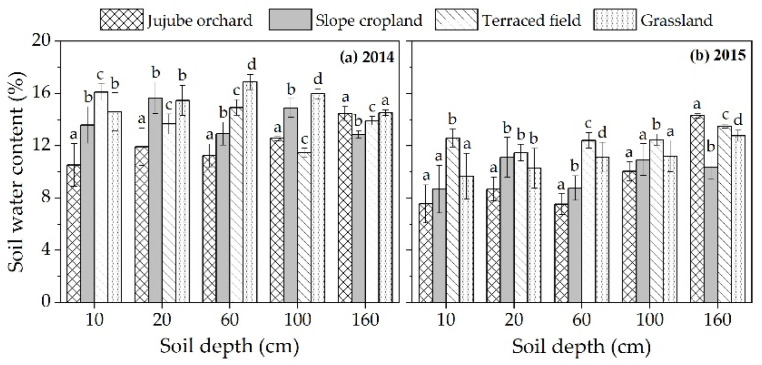
Vertical distribution of average soil water in different land uses during the growing seasons of 2014 (**a**) and 2015 (**b**). The soil water content for each land use type in the soil profile is the average of the daily mean water content for that soil layer throughout the growing season. Different lowercase letters in the same column represent significant differences between various land types within the same soil depth.

A comprehensive analysis of soil water content in each soil layer under different land use types during the 2014–2015 growing seasons revealed that land use type had a significant effect (*p* < 0.05) on soil water content in the 0–10 cm soil layer, with terraced field (14.4%) > grassland (12.1%) > slope cropland (11.1%) > jujube orchard (9.0%) (Figure 4). Jujube orchard contained significantly less soil water than the other three land types in the 10–20 cm soil layer (*p* < 0.05). Grassland and terraced field had a higher soil water content in the 20–60 cm soil layer than slope cropland and jujube orchard (*p* < 0.05). In the 60–100 cm soil layer, the water content of jujube orchard soil was significantly lower than slope cropland and grassland (*p* < 0.05). In the 100–160 cm soil layer, slope cropland had significantly less water content than other land uses (*p* < 0.05). In both growth seasons, the soil water content peaked at a depth of 20 cm in slope cropland but was as low as 160 cm in jujube orchards. In 2014, the greatest soil water content in terraced field and grassland was found at a depth of 10 cm and 60 cm, respectively, however in 2015, both soil water contents peaked at a depth of 160 cm. Due to the fact that soil water was governed by precipitation, plant cover (which influenced soil evaporation), and root dispersion, the position of peak soil water content varied across land use types. In years with plentiful precipitation, soil water was abundant in the shallow layer of terraced field and grassland, and the peak soil water location was upward; in years with little precipitation, the peak soil water location was downward. In 2014–2015, the valley position of soil water content in slope cropland and grassland rose from 160 cm to 10 cm, with respective valleys of 12.9% and 8.7% in slope cropland and 14.5% and 9.7% in grassland. The position of the valley value of soil water content in the terraced field grew from 100 cm to 20 cm, although at a slower rate, while the valley values remained relatively stable at around 11.5%. The valley value of the soil water content in the jujube orchard decreased from the top layer of 10 cm (10.5%) to the 60 cm depth (7.5%), which was close to the wilting point (7%), indicating that in dry years, the soil water in the shallow layer was insufficient to meet the transpiration water consumption demand of jujube trees. As a result, jujube plants began to absorb deeper soil water, leading deep soil water to reach the wilting point and forming a moderately dry layer (soil water content of 6–9%) [26].

### 3.3. Grey Relational Analysis of Soil Water at Various Depths for Different Land Use Types

To gain insight into the profile characteristics of soil water under varied land use practices in the research region, the grey relational analysis method was employed to assess the grey relational grade of soil water for distinct land use types and soil depths. The soil profiles were separated into three layers: the top layer (0–20 cm), the middle layer (20–100 cm), and the deep layer (100–160 cm), with the mean soil water content series of each layer from May to October set as *X*_1_ = {*X*_1_(*k*)|*k* = 5, 6, ..., 10}, *X*_2_ = {*X*_2_ (*k*)|*k* = 5, 6, ..., 10}, and *X*_3_ = {*X*_3_(*k*)|*k* = 5, 6, ..., 10}, respectively. The grey relational grade was determined using Equations (5) and (7), with the top layer serving as the reference series, the middle layer and the deep layer serving as the comparison series (denoted as *R*_12_ and *R*_13_, respectively), and the middle layer serving as the reference series and the deep layer serving as the comparison series (denoted as *R*_23_), respectively.

Similar patterns were seen in slope cropland, terraced field, and jujube orchard; the correlations between soil water in the top and middle layers were the closest, with the relationship with the deep layer coming in second (Table 2). This suggests that the majority of the soil water in the middle layer under these three land use types originated from surface seepage, as the middle layer was closest to the surface layer and precipitation-produced surface seepage reached the middle layer (100 cm) directly, while only a small portion reached the deep layer (below 100 cm). The correlation between the soil water in the deep layer and that in the middle layer was substantial, and it was also stronger than the correlation between the deep layer and the top layer. This is because the soil water in the deep layer primarily came from the secondary seepage of soil water in the middle layer, and only a small portion came from the surface seepage; the evaporation consumption of soil water in the deep layer must first pass through the middle layer and then to the top layer; and the infiltration amount and depth of precipitation decreased as soil depth increased. In addition, the highest correlation between the top and middle layers demonstrated that primary percolation is preferred to secondary percolation for soil water. The grey relational grade between the top layer and the deep layer in grassland did not vary substantially from that between the middle layer and the deep layer, suggesting that primary and secondary percolation were equivalent for deep soil water under the land use practice of wild grasses. Under diverse land uses, the changing pattern of soil water in the top layer was most comparable to that in the middle layer, with terraced field, followed by grassland, and slope cropland and jujube orchard having the least similarity. This shows that surface percolation under various land uses had differing degrees of supplementing of the soil water in the middle layer. The degree of proximity of soil water change dynamics between the top layer and deep layer, middle layer and deep layer was weaker than that between the top layer and middle layer, while the degree of proximity was fairly good in terraced field and relatively poor in jujube orchard, indicating that, compared to jujube orchard, the land use mode of terraced field had relatively deep regulation depth on soil water, and the change of soil water in the vertical direction was relatively stable. It may be extrapolated that various land use patterns have diverse effects on the developmental dynamics of soil water variations in distinct soil layers. This might be a result of the variable distribution of root depth and density in the soil for various plant kinds. For instance, the vegetation of grassland is herbaceous, the root distribution is shallow, and the depth of soil water utilization is limited, whereas the jujube tree, which is the principal tree species for soil and water conservation in loess hilly areas, has deep root distribution and high-water consumption. There are apparent changes in the grey relational grade due to the impact of the aforementioned variables on soil moisture trends at various soil depths. The terraces modify the original topography and slope, enhance precipitation penetration, and increase soil water, allowing soil water in deep layers to be continually replenished by soil moisture in shallow layers. However, owing to long-term clean tillage, the jujube orchard has exposed soil surface, severe soil compaction, limited soil porosity, and poor precipitation infiltration rate and amount, which is not favorable to the storage of rainfall and its penetration into the deep soil layer.

### 3.4. Soil Water Storage and Deficits for Different Types of Land Use

**Figure 5:**
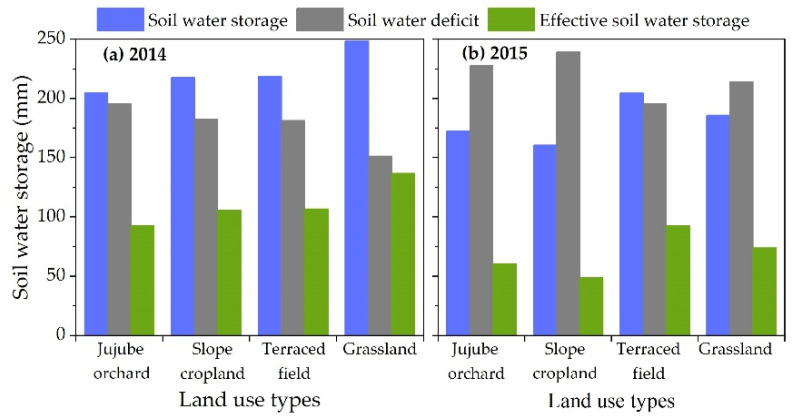
Average soil water storage, soil water deficit, and effective soil water storage in the 0–160 cm soil layer during the 2014 (**a**) and 2015 (**b**) growing seasons for various land uses.

Throughout the 2014 growing season, grassland had the maximum soil water storage in the 0–160 cm soil layer (up to 250 mm), followed by terraced field and jujube orchard (44 mm less than grassland) (Figure 5a). 2015 was a dry year in the study region, and soil water storage declined substantially across all land types (Figure 5b). The 0–160 cm soil layer of terraced field had a soil water storage of about 204 mm, which was greater than slope cropland, jujube orchard, and grassland by 43.90, 32.08, and 18.69 mm, respectively. Throughout the year, all land types had varied degrees of soil water deficit, with slope cropland and jujube orchard being the most affected. In 2015, the effective water storage in the 0–160 cm soil layer remained below 60 mm, representing 30.3% and 35.0%, respectively, of the total soil water storage. Due to the terraced field’s high water retention capacity, its soil water deficit was minimal, and the effective soil water storage accounted for 45.2% of the total water storage even in a drought year.

## 4. Discussion

### 4.1. The Effect of Various Land Uses on Soil Water

In dry and semiarid loess hilly regions, precipitation is the key factor determining the spatial and temporal variation of soil water. Combining the rainfall distribution in Figure 2 with the dynamics of soil water content over time in Figure 3, it can be determined that rainfall strongly influenced the soil water content in the shallow layer (0–60 cm) under each land use type, and that the soil water content in this layer rose rapidly after effective rainfall, with substantial soil water content changes. It has been demonstrated that land use and vegetation cover changes affect soil water movement by altering the nature of the substrate and the redistribution process of rainfall [27], i.e., land use type is a significant determinant of soil water distribution patterns, as supported by numerous studies [12,28,29]. In this study, land use types differed in their temporal dynamics, water storage characteristics, and vertical distribution of soil water under equal meteorological conditions (Figure 3, Figure 4 and Figure 5). 2015 was a dry year in the research location, and terraced fields revealed a strong ability to retain water. During the growth season, the 0–60 cm soil layer had considerably greater soil water content (*p* < 0.05) than the other three land types (Figure 3a). The average soil water content was 2.6%, 4.2%, and 1.8% greater than slope cropland, jujube orchard, and grassland, respectively (Figure 3a). The water storage capacity of the 0–160 cm soil layer was 43.90 mm, 32.08 mm, and 18.69 mm more than that of slope cropland, jujube orchard, and grassland, respectively (Figure 5b). During the 2014–2015 growth seasons, a high degree of consistency in the dynamics of soil water variation in all layers under the terraced field indicated that the vertical fluctuation of soil water in the terraced field was limited (Table 2). This is mostly due to the terraced field reducing the ground slope, modifying the original mild topography, generating a level field surface, avoiding runoff, and achieving the objectives of storing rainwater, enhancing infiltration, and improving soil water conditions through interception [7,30,31,32]. In addition to the kind of landform, the characteristics of the vegetation also have a significant role in influencing soil water variations. As the first link that influences the entry of precipitation into the soil, canopy interception influences the redistribution of precipitation. Studies have shown that tree canopies may absorb precipitation produced by minor precipitation events, hence leaving the soil water dynamics essentially independent of minor precipitation [33]. During the 2014–2015 growing seasons, over 70% of rainfall in the research region was ineffective (rainfall less than 5 mm) [34] (Figure 2) and was easily collected by jujube canopies for evaporation and unable to nourish the soil. In addition, jujube trees were widely spaced apart, and the bulk of the soil surface was bare, resulting in a high rate of soil evaporation. In 2014, the soil water content of the 0–60 cm soil layer in jujube orchard was 2.9%, 3.8%, and 4.5% lower than slope cropland, terraced field, and grassland, respectively (*p* < 0.05); in 2015, the soil water content of the 0–60 cm soil layer during the full fruit period was even lower than the wilting point, thereby forming a seasonal low moisture zone (Figure 3a). In the dry year, the soil water deficit in the jujube orchard was very severe, with the effective water storage in the 0–160 cm soil layer being less than 60 mm, representing only 35.0% of the total soil water storage (Figure 5b). During the 2014–2015 growth seasons, soil water dynamics in the different layers of jujube orchard were very variable (Table 2), showing inadequate vertical management of soil water. Soybean and natural grasses, due to their short plants, had a less pronounced canopy interception effect than jujube trees, could use more slope runoff than jujube trees, and dense vegetation covered the ground surface, reducing soil evaporation; thus, even though the slopes were the same and the difference in slope gradient was not statistically significant, the soil water status of slope cropland and grassland was superior to that of jujube orchard.

To further explore the changes in soil water distribution after precipitation for different land use types, maximum rainfall in the research region during the experiment was utilized as an example. Between 8 July and 11 July 2014, there was a total of 90.4 mm of rain. Effective precipitation was absent six days earlier (2 July to 7 July) and nine days later (12 July to 20 July). After one day of this rainfall, the soil water increment of 0–100 cm soil layer of slope cropland, terraced field, and grassland accounted for 99.9%, 99.5%, and 99.6%, respectively, of the total increase, while the 0–60 cm soil layer of jujube orchard accounted for 99.3% of the total increase, showing that this rainfall primarily replenished soil water in the 0–100 cm soil layer of slope cropland, terraced field, and grassland, and the 0–60 cm soil layer of jujube orchard. In other words, under identical rainfall conditions, the depth of rainfall infiltration in the jujube orchard was lower than that in the slope cropland, terraced field, and grassland. This difference could be attributed to the jujube tree canopy and branches’ rain-interception and buffering actions, which reduced the amount and velocity of rainfall reaching the surface and impacted rainfall infiltration. After five days of rain, the infiltration layer of the jujube orchard had a soil water loss rate of 15.6%, which was much greater than the terraced field’s rater of 3.9%. The rates for the slope cropland and grassland, however, were similar at 8.7% and 8.5%. The soil water loss rates of the slope cropland, terraced field, jujube orchard, and grassland were 15.0%, 6.9%, 30.9%, and 14.5%, respectively, after nine days of this rainfall., The soil water loss rate in terraced fields rose more slowly than in jujube orchards when compared to the soil water loss rate after five days of rainfall. This suggests that the terraced field had a significant water retention impact in persistently dry circumstances after the rains. In contrast, the soil water in the jujube orchard was severely lost, which may be related to the jujube orchard’s high evapotranspiration.

### 4.2. Suggestions for Water Management for Different Land Use Types

In loess hilly areas, soil water is a critical constraining factor in the recovery and rebuilding of vegetation and a fundamental regulator of land production. Soil water depletion and soil desiccation will intensify in the absence of scientific guidance on land use and resource allocation, eventually affecting ecological restoration and the sustainable use of land resources. Jujube trees can only grow healthily if the soil water level is maintained at more than 60% of the field capacity, according to many studies [35]. Figure 3a shows that, aside from two instances from the 9th to the 14th of July 2014 and the 28th to the 30th of September 2014, when it was higher than 15% due to the supplementation of heavy rainfall, the soil water content of the dense root layer (0–60 cm) in the jujube orchard was below 15% during the growing seasons of 2014–2015 and did not satisfy the water needs of the jujube trees. The fact that soil water in the 60–160 cm soil layer consistently stayed below 15% throughout the growth period (Figure 3b) demonstrates that jujube trees had difficulty meeting their own water demands by drawing from the deep soil layers. The growth and development of jujube trees will be negatively impacted if soil water scarcity is allowed to persist, which will finally result in the degeneration and degradation of jujube orchards. Therefore, it is crucial to implement soil water management practices in rain-fed jujube orchards, such as pruning jujube trees in a water-efficient way and maintaining tree specifications within a reasonable range, both of which can reduce crown interception and transpiration, thereby lowering excessive soil water consumption by jujube trees [36]. Additionally, to absorb part of the runoff from rainfall and increase the water content of the soil, horizontal ditches and fish-scale pits can be constructed in the jujube orchard [37,38,39]. The ineffective water consumption caused by soil evaporation may be reduced simultaneously by using soil water conservation strategies, including mulching with straw, film, and jujube branch cutting for local coverage [40,41,42].

Terraced fields exhibited much larger soil water contents than slope cropland during dry years, both in the dense root layer (0–60 cm) and the sparse root layer (60–160 cm) (*p* < 0.05) (Figure 3). This is primarily due to the fact that rainfall in the loess hilly region consists primarily of high-intensity rainstorms, and slope cropland is prone to produce more ineffective runoff, whereas terraces store more water than slope cropland and have a greater infiltration depth under the same rainfall intensity, the slope cropland may produce runoff, whereas the terraced field does not produce runoff throughout the entire process [43]. Some researchers have discovered that terraced fields can nearly entirely collect rainfall larger than 100 mm but less than 200 mm without creating runoff, based on monitoring data from various places on the Loess Plateau [44]. Although runoff will be produced as rainfall intensity increases, terraces can increase the infiltration of rainwater through multiple interception, significantly improving the soil water environment. This is because the flow velocity of runoff passing through the slope of terraced fields will be reduced and the confluence time will be prolonged. As a result, the authors contend that slope cropland to terraced field conversion, which intercepts natural precipitation, reduces surface runoff, increases soil infiltration, and improves precipitation utilization, is of the utmost importance in guiding the management and development of slope cropland and developing ecological agriculture in the loess hilly region.

## 5. Conclusions

The 0–60 cm soil layer under each land use type was the seasonal soil water fluctuation layer, whereas the 60–160 cm soil layer had little variance. During the 2014 growing season, the 0–60 cm soil layer of jujube orchard had 2.9%, 3.8%, and 4.5% less soil water than slope cropland, terraced field, and grassland, respectively (*p* < 0.05). Terraced fields had greater soil water content in each layer from 0 to 160 cm than the other three land types during the 2015 growing season (*p* < 0.05).

The jujube orchard had a significant soil water deficit, with the 0–160 cm soil layer storing just 35.0% of the total soil water storage during the dry year. The terraced field has 43.90, 32.08, and 18.69 mm greater soil water storage than slope cropland, jujube orchard, and grassland in the dry year.

The four land use types showed comparable soil water changes in the top layer (0–20 cm) and middle layer (20–100 cm). The order of similarity was terraced field > grassland > slope cropland > jujube orchard. Except for grassland, soil water variations in the top layer and deep layer (100–160 cm) were different under other land uses.

Water management measures should be used to limit transpiration and other wasteful water usage by jujube trees in loess hilly areas to promote sustainable growth and prevent decay. Terraced fields have far better soil water conditions than slope croplands. Therefore, changing slope croplands to terraced fields will improve rainfall usage.

## Figures and Tables

**Table 1:** The basic physical properties of the soil in the experimental plots.

Land Use Type	Soil Layer (cm)	Soil Texture			Bulk Density (g·cm^−3^)	Soil Porosity (%)	Saturated Hydraulic Conductivity (cm·d^−1^)
		Silt (%)	Sand (%)	Clay (%)			
Jujube orchard	0–20	62.6 ± 1.9	23.7 ± 4.0	13.7 ± 2.4	1.31 ± 0.12	51.7 ± 4.5	36.6 ± 9.6
	20–40	64.0 ± 1.9	21.6 ± 3.4	14.4 ± 2.7	1.41 ± 0.10	52.1 ± 3.8	
	40–60	64.2 ± 1.2	20.7 ± 2.2	15.1 ± 2.5	−	−	
Slope cropland	0–20	63.0 ± 3.7	21.0 ± 5.6	16.0 ± 3.9	1.17 ± 0.15	58.9 ± 5.6	74.2 ± 20.6
	20–40	63.4 ± 2.4	19.5 ± 5.4	17.0 ± 4.5	1.29 ± 0.11	54.3 ± 3.7	
	40–60	65.0 ± 1.9	19.9 ± 3.9	15.1 ± 3.2	−	−	
Terraced field	0–20	63.8 ± 2.3	17.7 ± 1.9	18.5 ± 2.9	1.26 ± 0.11	55.1 ± 4.2	55.6 ± 10.4
	20–40	64.9 ± 1.7	16.6 ± 3.9	18.6 ± 4.1	1.36 ± 0.07	52.5 ± 2.7	
	40–60	63.3 ± 1.1	16.2 ± 4.1	20.5 ± 4.2	−	−	
Grassland	0–20	63.6 ± 1.3	17.3 ± 2.8	19.1 ± 3.4	1.28 ± 0.09	54.0 ± 3.4	35.3 ± 8.3
	20–40	62.9 ± 1.6	15.1 ± 1.7	22.0 ± 0.7	1.28 ± 0.04	52.1 ± 1.5	
	40–60	63.3 ± 1.3	14.9 ± 3.7	21.8 ± 3.8	−	−	

Note: The soil samples were collected on 5 September 2014. The data are expressed as mean ± standard deviation, *n* = 3. Soil particle composition: Sand% (0.02–2 mm), Silt% (0.002–0.02 mm), and Clay% (<0.002 mm).

**Table 2:** Grey relational grade of soil water in different layers under different land uses throughout the growing season.

Land Use Type	2014			2015		
	*R*_12_	*R*_13_	*R*_23_	*R*_12_	*R*_13_	*R*_23_
Jujube orchard	0.67	0.52	0.59	0.60	0.46	0.45
Slope cropland	0.68	0.59	0.64	0.63	0.57	0.60
Terraced field	0.77	0.70	0.75	0.70	0.68	0.69
Grassland	0.75	0.67	0.67	0.69	0.62	0.61

Note: *R*_12_ is the grey relational grade, whereby the top layer is the reference series, and the middle layer is the comparison series. *R*_13_ is the grey relational grade, having the reference series in the top layer and the comparison series in the deep layer. Lastly, *R*_23_ denotes the grey relational grade, whereby the middle layer serves as the reference series and the deep layer serves as the comparison series.

## Data Availability

Not applicable.
